# Input Forces Estimation for Nonlinear Systems by Applying a Square-Root Cubature Kalman Filter

**DOI:** 10.3390/ma10101162

**Published:** 2017-10-10

**Authors:** Xuegang Song, Yuexin Zhang, Dakai Liang

**Affiliations:** 1 State Key Laboratory of Mechanics and Control of Mechanical Structures, Nanjing University of Aeronautics and Astronautics, Nanjing 210016, China; sxg37068219890209@126.com; 2 Key Laboratory of Micro-Inertial Instrument and Advanced Navigation Technology, Ministry of Education, School of Instrument Science and Engineering, Southeast University, Nanjing 210096, China; smileyuexin@163.com

**Keywords:** input forces estimation, nonlinear algorithm, square-root cubature Kalman filter, nonlinear estimator

## Abstract

This work presents a novel inverse algorithm to estimate time-varying input forces in nonlinear beam systems. With the system parameters determined, the input forces can be estimated in real-time from dynamic responses, which can be used for structural health monitoring. In the process of input forces estimation, the Runge-Kutta fourth-order algorithm was employed to discretize the state equations; a square-root cubature Kalman filter (SRCKF) was employed to suppress white noise; the residual innovation sequences, a priori state estimate, gain matrix, and innovation covariance generated by SRCKF were employed to estimate the magnitude and location of input forces by using a nonlinear estimator. The nonlinear estimator was based on the least squares method. Numerical simulations of a large deflection beam and an experiment of a linear beam constrained by a nonlinear spring were employed. The results demonstrated accuracy of the nonlinear algorithm.

## 1. Introduction

Advanced structural health monitoring is generally regarded as a vital technology for the next generation of aeronautical and space systems [1]. This technology is aimed at preventing catastrophic structural failures and is comprised of three facets: (a) determination of stresses and deformations of structural components; (b) estimating input forces; and (c) detection of critical damage mechanisms such as cracking, delamination, and corrosion. Estimating dynamic input forces of engineering structures accurately has great significance for structural health monitoring, fatigue analysis and life estimation, and is regarded as the premise of structural design and optimization. Damage tolerance considerations determine the design of composite components, and forces occurring in service conditions can induce non-visible damages in structures. Besides, additional uncertainties may arise, as structural architectures behave in complex ways under the action of thermo-mechanical forces. By knowing forces distribution, we can adjust structure layout and enhance material performance to ensure safety. Moreover, the presence of damage can modify load paths in difficulty predictable ways. By knowing force distribution, we can also assess damage as there is usually structural damage in the location of the forces mutation. In engineering applications, input forces are quite hard or even impossible to be measured directly. Some examples are the measurements of airplane wing deflection, blade shape changes of windmill or helicopters, tool-tip displacement of line boring machines, etc. In these cases, it is helpful to use attached sensors to measure the responses of structures. Estimating dynamic input forces by using responses of structures is an inverse problem. In mathematics, inverse problems are typically ill-posed and are difficult to solve. For inverse problems, a little measurement errors can cause large estimation errors. So input forces estimation method is the key to improve accuracy.

In summary, input forces estimation algorithms [2,3,4,5,6,7] can be divided into time domain algorithms and frequency domain algorithms. Time domain algorithms are much more valuable than frequency domain algorithms, as time domain algorithms can accomplish estimation in real-time. In dealing with nonlinear beam systems, there is real-time change in the stiffness matrix, which means that frequency domain algorithms cannot be used at this situation. In the process of the input forces estimation, suppressing noise is the key to improve accuracy, as small measurement errors can cause large estimation errors in inverse problems. For most practical engineering applications, noises can be simplified as white Gaussian noise. This means that the method of suppressing white Gaussian noise need to be studied first. Ma et al. [8,9] proposed a method to estimate input forces of linear beam systems by combining Kalman filter with a recursive method. In his work, Kalman filter was used to suppress noise, and residual innovation sequences, a priori state estimate, and innovation covariance generated by Kalman filter were used to estimate input forces by using a least-squares method. However, these studies were associated with linear beam systems, and estimating dynamic input forces of nonlinear beam systems have not been studied. As nonlinear beam systems are widely used in engineering applications, this work focus on estimating input forces for nonlinear beam systems.

In the work, with the system parameters determined, the magnitude and location of input forces in nonlinear systems can be estimated by using SRCKF and a nonlinear estimator. The nonlinear estimator is based on least squares method. According to the second order dynamic system and measuring principle, the state equations and measurement equations of the state-space model are established. The Runge-Kutta fourth-order algorithm is employed to discrete the state equations. SRCKF is used to suppress noise, and the residual innovation sequences, a priori state estimate, gain matrix and innovation covariance generated by SRCKF are employed to estimate the magnitude and location of input forces by using a nonlinear estimator. To verify the effectiveness of this estimation method, numerical simulations of a large deflection beam and experiment of a linear beam constrained by a nonlinear spring are employed.

## 2. Problem Formulation

There are three steps to estimate the location and magnitude of input forces. First, the state equation and measurement equation of the state-space model are discretized by using a Runge-Kutta method. Second, SRCKF is used to suppress white noise. Finally, residual innovation sequences, a priori state estimate, gain matrix and innovation covariance generated by SRCKF are used to estimate the location and magnitude of input forces.

### 2.1. Discretization of a Nonlinear System

In the paper, the dynamic parameters of nonlinear beam structure were known, and we could then construct the state-space model. With unknown parameters of structure in engineering applications, we could estimate dynamic and static parameters of state-space models according to Refs. [10,11,12,13]. The Runge-Kutta methods are widely used to discrete continuous-time system, and the most well-known member Runge-Kutta fourth-order algorithm (RK4) has the advantages of high precision, convergence and stability. Therefore, RK4 is employed by the paper. The nonlinear, continuous-time model can be described by:

(1) $$\dot{X}\left(t\right)=f_{0}\left[X\left(t\right),F\left(t\right),t\right]+w\left(t\right)$$

(2) $$\dot{Z}\left(t\right)=h_{0}\left[X\left(t\right),t\right]+v\left(t\right)$$

where state vector *X*(*t*) = [position; velocity] and observation vector *Z*(*t*) represent measured dynamic responses; vector *F*(*t*) = [0; *F*]; $f_{0}$(·) and $h_{0}$(·) are nonlinear functions with respect to *X*, *F* and *t*; *F* is the forces vector; *w*(*t*) and *v*(*t*) represent continuous-time white noise processes.

For a step-size Δ*T* > 0 and an initial value $X_{k-1}$, the state Equation (1) can be discrete as follows:

$$X_{k}=X_{k-1}+\frac{\Delta T}{6}(b_{1}+2\times b_{2}+2\times b_{3}+b_{4})$$

for *k* = 1, 2, 3, 4, …, using:

$$\left\{ \begin{array}{l} b_{1}=f_{0}[X_{k-1},F_{k-1},t_{k-1}]\\ b_{2}=f_{0}[X_{k-1}+\frac{b_{1}}{2},F_{k-1},t_{k-1}+\frac{\Delta T}{2}]\\ b_{3}=f_{0}[X_{k-1}+\frac{b_{2}}{2},F_{k-1},t_{k-1}+\frac{\Delta T}{2}]\\ b_{4}=f_{0}[X_{k-1}+b_{3},F_{k-1},t_{k-1}+\Delta T] \end{array}\right.$$

Here, $X_{k}$ is the state approximation, and the value $X_{k}$ is determined by the value of $X_{k-1}$ plus the weighted average of the increments ($b_{1}$, $b_{2}$, $b_{3}$, and $b_{4}$), where each increment is the product of the sample interval.

The discrete model is described by:

(3) $$\left\{ \begin{array}{l} X_{k}=f(X_{k-1},F_{k-1})+w_{k}\\ Z_{k}=h(X_{k})+v_{k} \end{array}\right.$$

$E\left[w_{k}\right]=0,\;E\left[w_{k}w_{l}^{T}\right]=Q\delta_{kl},\;Q=Q_{w}I_{2n*2n}$, where vector $w_{k}$ represents the process white noise, *Q* represents covariance matrix and $\delta_{kl}$ is the Kronecker deltas. $E\left[v_{k}\right]=0$, $E\left[v_{k}v_{l}^{T}\right]=R\delta_{kl},\;R=R_{v}I_{2n*2n}$, where vector $v_{k}$ represents the measurement white noise, *R* represents the noise covariance matrix, $R_{v}=\sigma^{2}$, *σ* is the standard deviation of the measurement noise. The vectors $w_{k}$ and $v_{k}$ are mutually uncorrelated.

### 2.2. Square-Root Cubature Kalman Filter [14,15]

#### 2.2.1. Initialization

Initialization the Filter by Setting Initiate State and Square Root of Covariance Matrix.

(4) $${\hat{x}}_{0|0}=E[x_{0}]$$

(5) $$S_{0|0}=chol[(x_{0}-{\hat{x}}_{0|0}){\left(x_{0}-{\hat{x}}_{0|0}\right)}^{T}]$$

The initial value $S_{0|0}$ of the square-root factor of the error covariance matrix can be computed by the Cholesky decomposition, where *chol*[·] is the Cholesky factorization.

#### 2.2.2. Time Update

(1) Calculate the cubature points:

(6) $$X_{i,k/k}=S_{k/k}\xi_{i}+{\hat{x}}_{k/k},\;i=1,2,\ldots ,m$$

where ${\hat{x}}_{k/k}$ is the prior estimated state. $\xi_{i}=\sqrt{\frac{m}{2}}{\left[1\right]}_{i}$, ${\left[1\right]}_{i}$ is the *i*th column of the matrix [***I*** (−1)***I***].

(2) Calculate the propagated cubature points:

(7) $$X_{i,k+1/k}^{*}=f_{k}\left(X_{i,k/k}\right),\;i=1,2,\ldots ,m$$

(3) Calculate the predicted state:

(8) $${\hat{x}}_{k+1/k}=\frac{1}{m}\sum_{i=1}^{m}X_{i,k+1/k}^{*}$$

(4) Calculate the square-root factor of prediction error covariance:

$$S_{k+1/k}=tria\left(\left[X_{k+1/k}^{*},S_{Q,k}\right]\right)$$

where $S_{Q,k}$ is obtained by Cholesky factorization for $Q_{k}=S_{Q,k}S_{Q,K}^{T}$. tria(⋅) is a matrix triangularization algorithm which can generate a lower triangular matrix:

(9) $$X_{k+1/k}^{*}=\frac{1}{\sqrt{m}}\left[X_{1,k+1/k}^{*}-{\hat{x}}_{k+1/k},X_{2,k+1/k}^{*}-{\hat{x}}_{k+1/k},\cdots ,X_{m,k+1/k}^{*}-{\overset{}{\hat{x}}}_{k+1/k}\right]$$

#### 2.2.3. Measurement Update

(1) Calculate the cubature points:

(10) $$X_{i,k+1/k}=S_{k+1/k}\xi_{i}+{\hat{x}}_{k+1/k},\;i=1,2,\cdots ,m$$

(2) Calculate the propagated cubature points:

(11) $$Y_{i,k+1/k}=h_{k+1}\left(X_{i,k+1/k}\right),\;i=1,2,\cdots ,m$$

(3) Calculate the predicted state:

(12) $${\hat{z}}_{k+1/k}=\frac{1}{m}\sum_{i=1}^{m}Y_{i,k+1/k}$$

(4) Calculate the square-root of the innovation covariance matrix:

(13) $$S_{zz.k+1/k}=tria\left(\left[Y_{k+1/k},S_{R,k+1}\right]\right)$$

$Y_{k+1/k}^{}=\frac{1}{\sqrt{m}}\left[Y_{1,k+1/k}^{}-{\hat{z}}_{k+1/k},Y_{2,k+1/k}^{}-{\hat{z}}_{k+1/k},\cdots ,Y_{m,k+1/k}^{}-{\hat{z}}_{k+1/k}\right]$, $S_{R,k+1}$ is obtained by Cholesky factorization for $R_{k+1}=S_{R,k+1}S_{R,k+1}^{T}$ and $R_{k+1}$ is the noise covariance matrix. tria(⋅) is a matrix triangularization algorithm which can generate a lower triangular matrix.

(5) Calculate the innovation covariance matrix:

(14) $$P_{zz,k+1/k}=S_{zz,k+1/k}S_{zz,k+1/k}^{T}$$

(6) Calculate the cross-covariance matrix:

(15) $$P_{xz,k+1/k}=X_{k+1/k}Y_{k+1/k}^{T}$$

where

$$X_{k+1/k}=\frac{1}{\sqrt{m}}\left[X_{1,k+1/k}-{\hat{x}}_{k+1/k},X_{2,k+1/k}-{\hat{x}}_{k+1/k},\cdots ,X_{m,k+1/k}-{\hat{x}}_{k+1/k}\right]$$

(7) Calculate the Kalman gain:

(16) $$K_{k+1}=\left(P_{xz,k+1/k}/S_{zz,k+1/k}^{T}\right)/S_{zz,k+1/k}$$

(8) Calculate the updated state:

(17) $${\bar{Z}}_{k+1}=z_{k+1}-{\hat{x}}_{k+1/k}$$

(18) $${\hat{x}}_{k+1/k+1}={\hat{x}}_{k+1/k}+K_{k+1}\bar{Z}(k+1)$$

(9) Calculate the square-root factor of the corresponding error covariance:

(19) $$S_{k+1/k+1}=tria\left(\left[X_{k+1/k}-K_{k+1}\times Y_{k+1/k},K_{k+1}S_{R,k+1}\right]\right)$$

tria(⋅) is a matrix triangularization algorithm which can generate a lower triangular matrix.

### 2.3. The Nonlinear Estimator

By applying residual innovation sequences, a priori state estimate, gain matrix and innovation covariance generated by SRCKF, input forces can be estimated by using a nonlinear estimator from the response values (displacement, velocity, or acceleration). The inverse estimation method consists of two parts: SRCKF with no input forces terms, and a nonlinear estimator. In the nonlinear estimator, the first-order Taylor series expansion is used to arrive at the estimated state value ${\hat{x}}_{k/k-1}$, and a least squares method is used to estimate forces. The detailed derivation of the nonlinear estimator can be found in Appendix A. The simple equations of the nonlinear estimator are as follows:

(20) $$\Phi_{k}=\partial f({\hat{X}}_{k/k-1})/\partial X$$

(21) $$\Gamma_{k}=\partial f({\hat{X}}_{k/k-1})/\partial F$$

(22) $$H_{k}=\partial h({\hat{X}}_{k/k-1})/\partial X$$

(23) $$B_{s}(k)=H_{k}[\Phi_{k}M_{s}(k-1)+I]\Gamma_{k}$$

(24) $$M_{s}(k)=[I-K_{k}H_{k}][\Phi_{k}M_{s}(k-1)+I]$$

(25) $$K_{b}(k)=\gamma^{-1}P_{b}(k-1)B_{s}{}^{T}(k){\left[B_{s}(k)\gamma^{-1}P_{b}(k-1)B_{s}{}^{T}(k)+P_{zz,k/k-1}\right]}^{-1}$$

(26) $$P_{b}(k)=[1-K_{b}(b)B_{s}(k)]\gamma^{-1}P_{b}(k-1)$$

(27) $$\hat{F}(k)=\hat{F}(k-1)+K_{b}(k)[{\bar{Z}}_{k}-B_{s}(k)\hat{F}(k-1)]$$

where *f*(·) and *h*(·) represent nonlinear functions of the discrete system, $P_{zz,k/k-1}$ represents the innovation covariance matrix, $K_{k}$ represents the gain matrix, $B_{s}\left(k\right)$ and $M_{s}\left(k\right)$ represent the sensitivity matrices, ${\bar{Z}}_{k}$ represents the innovation matrix, $K_{b}\left(k\right)$ represents the correction gain for updating $\hat{F}\left(k\right)$, $P_{b}\left(k\right)$ represents the error covariance, $\hat{F}\left(k\right)$ represents the estimated input vector, and *γ* is a fading factor.

**Figure 1:**
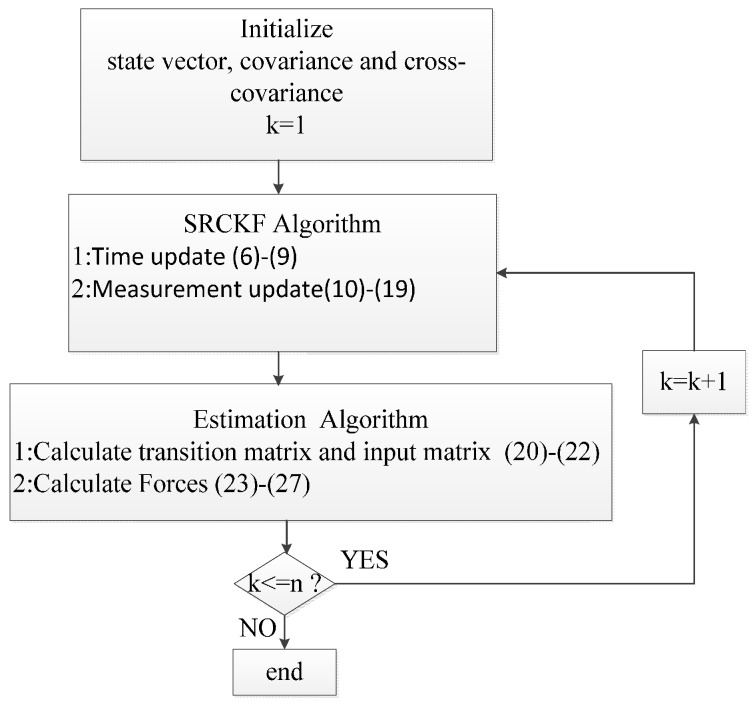
The flow chart of estimation method.

The procedures for the nonlinear method are summarized as Figure 1.

## 3. Numerical Simulations and Discussions

### 3.1. Simulation Model

In structural dynamic analysis, the slender beam may exhibit geometrically nonlinear behaviors when it undergo large deformation. In the paper, a large deformation beam is picked as the model, and the equation of motion can be described as follows:

(28) $$M\ddot{X}+C\dot{X}+K(X)X=F$$

where *M* is the mass matrix, *C* the damping matrix, *K* the stiffness matrix, *X* the displacement vector, and *F* the equivalent nodal force vector.

$$M=\sum_{i=1}^{N}M^{e}\;\;K=\sum_{i=1}^{N}K^{e}\;\;K^{e}=K_{L}{}^{e}+K_{N}{}^{e}$$

**Figure 2:**
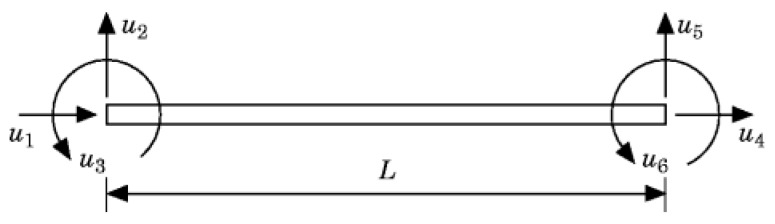
Beam element.

It is assumed that the beam element is two-dimensional and each node has three degrees of freedom (two translational and one rotational). Beam element is shown as Figure 2.

According to Refs. [16,17,18], the element linear stiffness matrix $K_{L}^{e}$, nonlinear stiffness matrix $K_{N}^{e}$ and consistent mass matrix $M^{e}$ can be expressed in the form:

$$\begin{array}{c} K_{L}^{e}=\frac{EI}{L^{3}}\left[\begin{array}{cccccc} AL^{2}/I & 0 & 0 & -AL^{2}/I & 0 & 0\\ & 12 & 6L & 0 & -12 & 6L\\ & & 4L^{2} & 0 & -6L & 2L^{2}\\ & & & AL^{2}/I & 0 & 0\\ Symmetric & & & & 12 & -6L\\ & & & & & 4L^{2} \end{array}\right]\\ K_{N}^{e}=\frac{EA(u_{4}-u_{1})}{L^{2}}\left[\begin{array}{cccccc} 0 & 0_{1} & 0 & 0 & 0 & 0\\ & 6/5 & L/10 & 0 & -6/5 & L/10\\ & & 2L^{2}/15 & 0 & -L/10 & -L^{2}/30\\ & & & 0 & 0 & 0\\ Symmetric & & & & 6/5 & -L/10\\ & & & & & 2L^{2}/15 \end{array}\right]\\ M^{e}=\frac{\rho AL}{420}\left[\begin{array}{cccccc} 140 & 0 & 0 & 70 & 0 & 0\\ & 156 & 22L & 0 & 54 & -13L\\ & & 4L^{2} & 0 & 13L & -3L^{2}\\ & & & 140 & 0 & 0\\ Symmetric & & & & 156 & -22L\\ & & & & & 4L^{2} \end{array}\right] \end{array}$$

where *ρ* is mass density, *A* is the area of cross-section, *L* is the beam element length, *E* is Young’s modulus of elasticity, *I* is the moment of inertia of the cross-section, and $u_{1}$ $u_{4}$ are the axial deformation of the first node and second node.

In converting the second order dynamic system to the state-space model, the state equation and measurement equation can be written as:

(29) $$\dot{Y}(t)=f(Y(t))+BF(t)$$

(30) Z(t)=HY(t)

where:

$$Y(t)=\left[\begin{array}{l} X(t)\\ \dot{X}(t) \end{array}\right],\;B=\left[\begin{array}{l} 0_{n\times n}\\ M^{-1} \end{array}\right]$$

The state value is $Y\left(t\right)={\left[Y_{1}\left(t\right),Y_{2}\left(t\right),\ldots ,\;Y_{3n-1}\left(t\right),Y_{3n}\left(t\right)\right]}^{T}$, and the forces value $F\left(t\right)={\left[F_{1},F_{2},F_{3},\ldots ,\;F_{3n}\right]}^{T}$. *f*(·) is a nonlinear function with respect to *Y*. H is a measurement matrix and Z(t) represents the measurement values vector.

Equations (29) and (30) are discretized using RK4, and the discrete model can be described by:

(31) $$\left\{ \begin{array}{l} Y_{k}=f(Y_{k-1},F_{k-1})+w_{k}\\ Z_{k}=h(Y_{k})+v_{k} \end{array}\right.$$

$Y_{k}$ is a state vector; $Z_{k}$ is a measurement values vector; *f*(·) and *h*(·) are nonlinear functions. $E\left[w_{k}\right]=0,\;E\left[w_{k}w_{l}^{T}\right]=Q\delta_{kl},\;Q=Q_{w}I_{2n*2n}$, where vector $w_{k}$ represents the process white noise, *Q* represents the covariance matrix, and $\delta_{kl}$ is the Kronecker deltas. $E\left[v_{k}\right]=0$, $E\left[v_{k}v_{l}^{T}\right]=R\delta_{kl},\;R=R_{v}I_{2n*2n}$, where vector $v_{k}$ represents the measurement's white noise, *R* represents the noise covariance matrix, $R_{v}=\sigma^{2}$, and *σ* is the standard deviation of the measurement noise. The vectors $w_{k}$ and $v_{k}$ are mutually uncorrelated.

Considering a five-element beam, the parameters of the beam are: Elastic modulus $E=7.2\times 10^{10}\;\left(N/m^{2}\right);$ density $\rho =2.7\times 10^{3}\;\left(\mathrm{kg}/m^{3}\right)$; beam length l=1 m; cross section S=0.1 m×0.01 m; sampling interval ΔT = 0.001 s. The system responses (displacements and rotations) are obtained by RK4, and the responses with white noise are employed as the measured dynamic responses. Thus, the magnitude and location of input forces can be estimated in real-time from dynamic responses (displacements and rotations).

The initial parameters of the estimation system are generally listed as follows: $x_{0}=0_{30\times 1}$, $P_{1}=I_{30\times 30}$, $P_{2}=0_{30\times 30}$, $M_{s}=200\times I_{30\times 30}$, $P_{b}=200\times I_{30\times 30}$, γ=0.69. To verify the effectiveness of this estimation method, the root-mean-square error (RMSE) method is used to measure the errors between the estimated forces ${\hat{F}}_{i}$ and the exact forces $F_{i}$. The RMSE is computed as follows:

(32) $$RMSE=\sqrt{\sum_{i=1}^{n}{\left(F_{i}-{\hat{F}}_{i}\right)}^{2}/n}$$

### 3.2. Simulation Results

**Figure 3:**
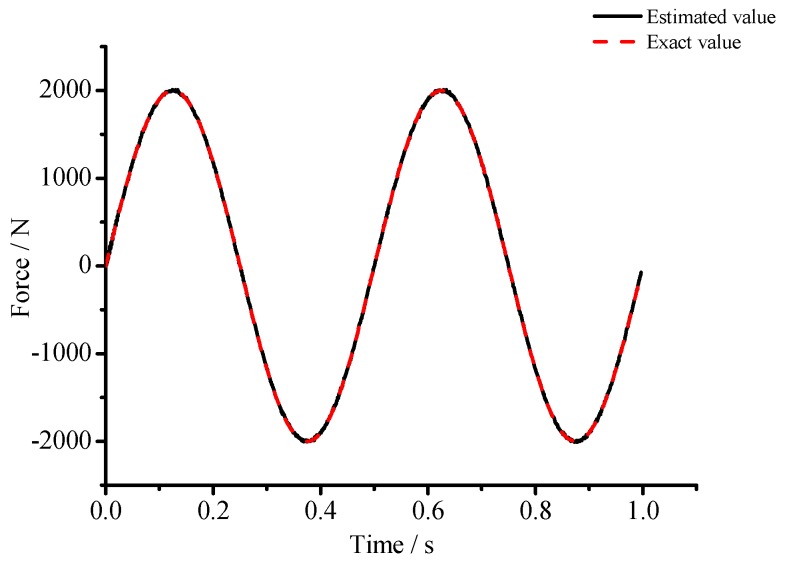
Estimation results of the sinusoidal input force (*Q_w_* = 1 × 10^−4^, *σ* = 1 × 10^−8^).

**Figure 4:**
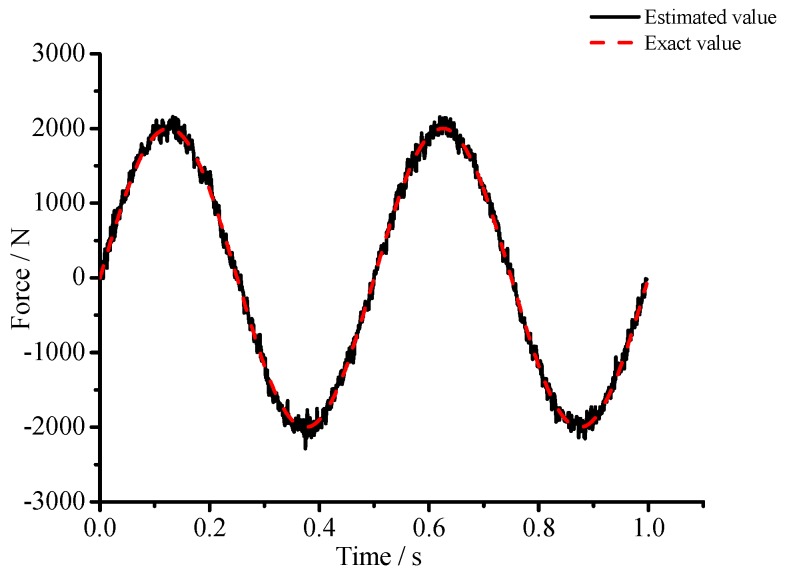
Estimation results of the sinusoidal input force (*Q_w_* = 1 × 10^−3^, *σ* = 1 × 10^−6^).

**Figure 5:**
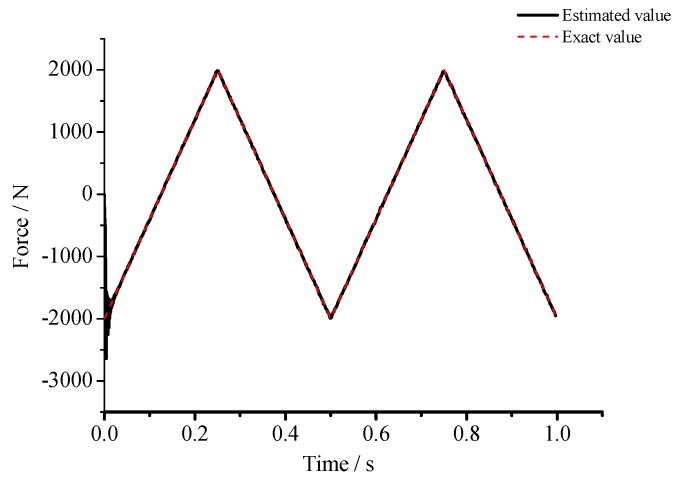
Estimation results of the triangular input force (*Q_w_* = 1 × 10^−4^, *σ* = 1 × 10^−8^).

**Figure 6:**
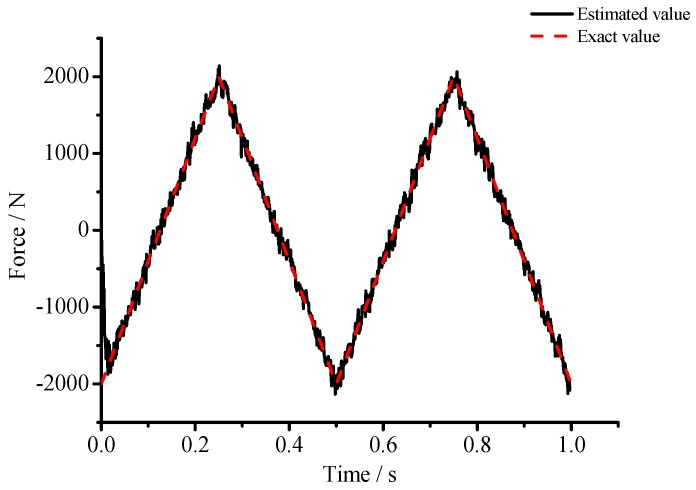
Estimation results of the triangular input force (*Q_w_* = 1 × 10^−3^, *σ* = 1 × 10^−6^).

**Figure 7:**
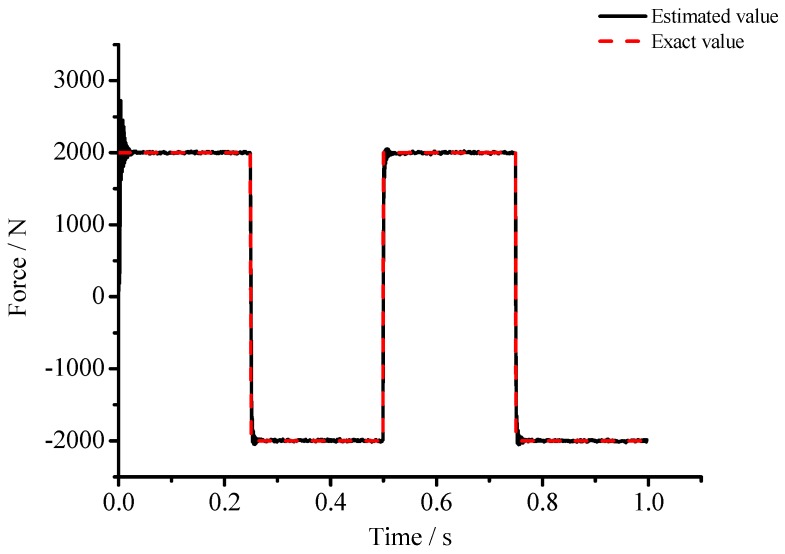
Estimation results of the rectangular input force (*Q_w_* = 1 × 10^−4^, *σ* = 1 × 10^−8^).

**Figure 8:**
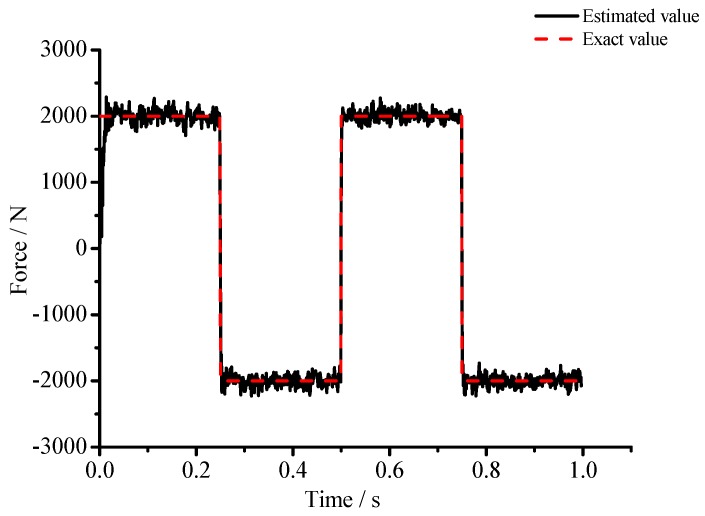
Estimation results of the rectangular input force (*Q_w_* = 1 × 10^−3^, *σ* = 1 × 10^−6^).

**Figure 9:**
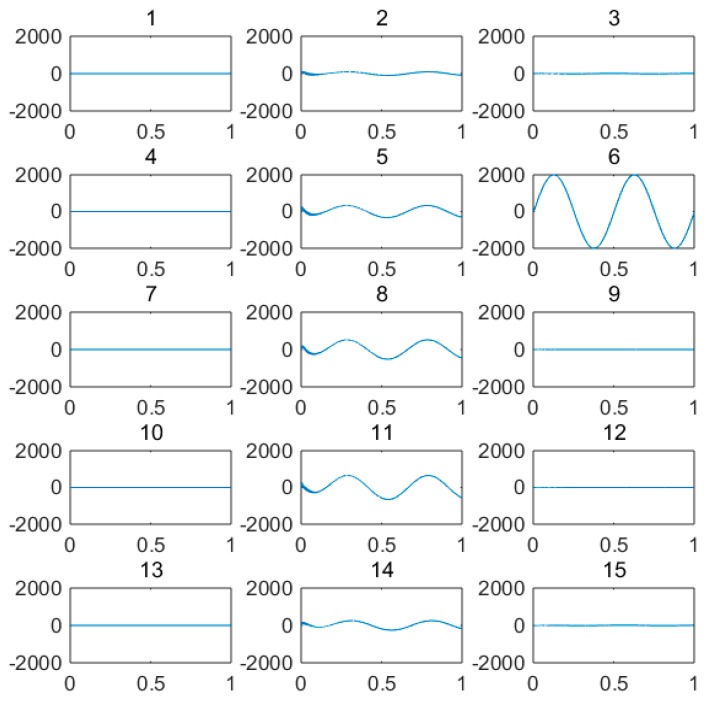
Location estimation of sinusoidal force (*Q_w_* = 1 × 10^−8^, *σ* = 1 × 10^−16^).

**Figure 10:**
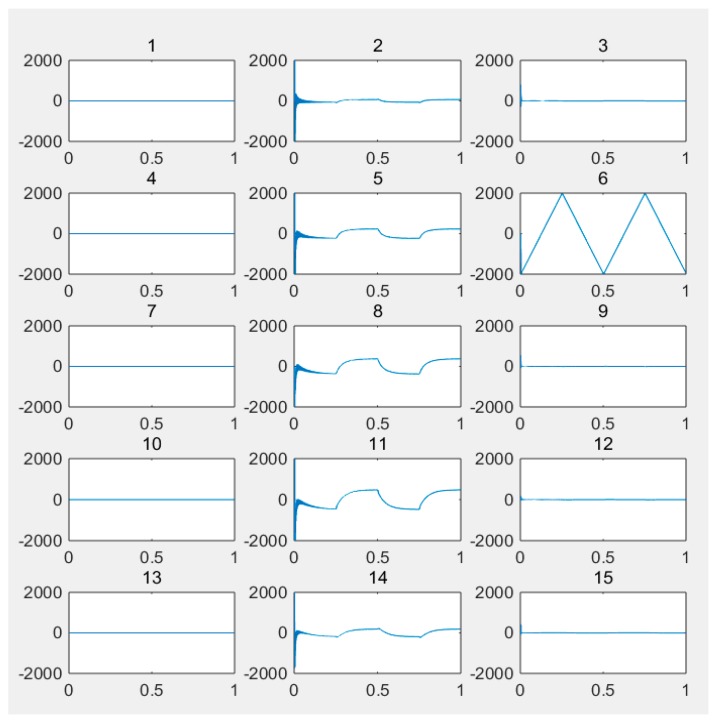
Location estimation of triangular force (*Q_w_* = 1 × 10^−8^, *σ* = 1 × 10^−16^).

**Figure 11:**
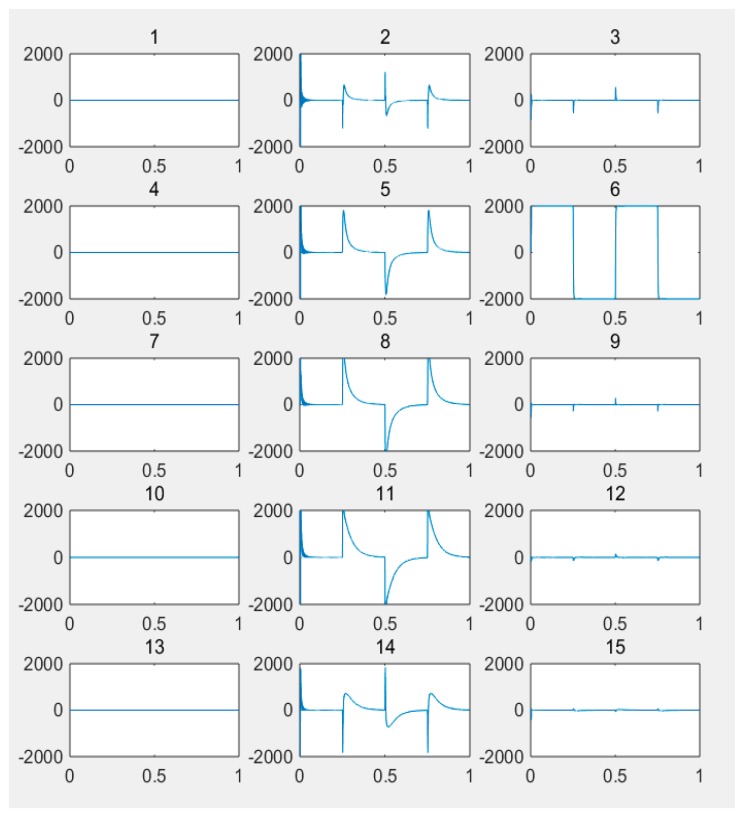
Location estimation of rectangular force (*Q_w_* = 1 × 10^−8^, *σ* = 1 × 10^−16^).

(1) For the large deflection beam model, a sinusoidal input force, a rectangular input force, and a triangular input force are estimated, respectively. Figure 3, Figure 4, Figure 5, Figure 6, Figure 7 and Figure 8 plot the results of magnitude estimation. For the method proposed in the paper, the forces of total degrees of freedom can be estimated, so we can estimate the location of force if single force is applied. In the simulation, force is applied at the sixth degree of freedom with a total of 15 degrees of freedom. The results of estimation of 15 forces at 15 degrees of freedom are plotted at Figure 9, Figure 10 and Figure 11.

(2) The performance (the mean errors and RMSE values) of estimation method with $Q_{w}$ at 1 × 10^−4^, 1 × 10^−6^ and σ at a constant 1 × 10^−8^ are presented in Table 1.

### 3.3. Discussions of the Simulations

(1) The SRCKF is famous for strong stability and higher precision, and only needs recent measurement data and the previous estimated value to estimate input forces, and so the proposed method could save computer memory, reduce computational burdens, and improve system robustness.

(2) Figure 5, Figure 6, Figure 7 and Figure 8 show that the estimated input forces rapidly converge to exact input forces with non-zero initial state, but with a large initial estimation errors. Table 1 show that the estimation performance of the sinusoidal input force are better than that of the rectangular input force and triangular input force, and this is because abrupt changes of input force will cause large errors. Figure 4, Figure 6 and Figure 8 show that the system have good stability with large system noise and measurement noise. From Figure 9, Figure 10 and Figure 11, we can conclude that the location estimation of forces have a good performance. For three types of forces, the estimation performance of the sinusoidal input force and the triangular input force are better than that of the rectangular input force.

(3) The results in Table 1 show that the proposed estimation method has good capabilities to suppress noise. The mean errors for sinusoidal input force, rectangular input force and triangular input force are close to zero. The RMSE values for sinusoidal input force, rectangular input force, and triangular input force are less than 0.46%, 6.61%, and 5.27%, respectively.

## 4. Experiment and Results

### 4.1. Experimental Model and Measurement Principle

**Figure 12:**
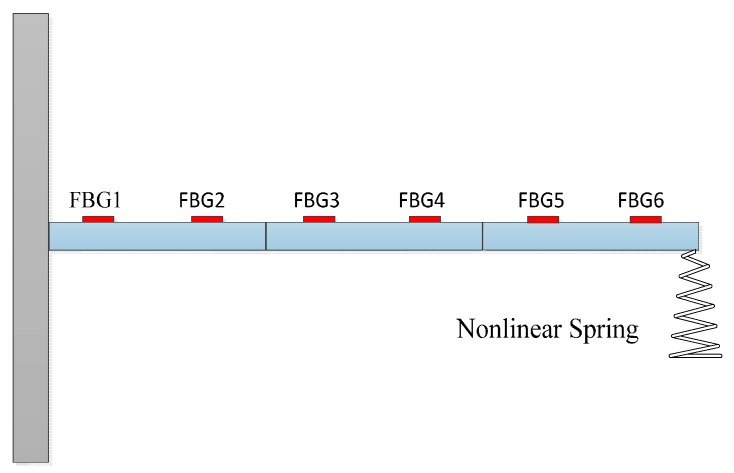
Beam model and FBG sensing network.

**Figure 13:**
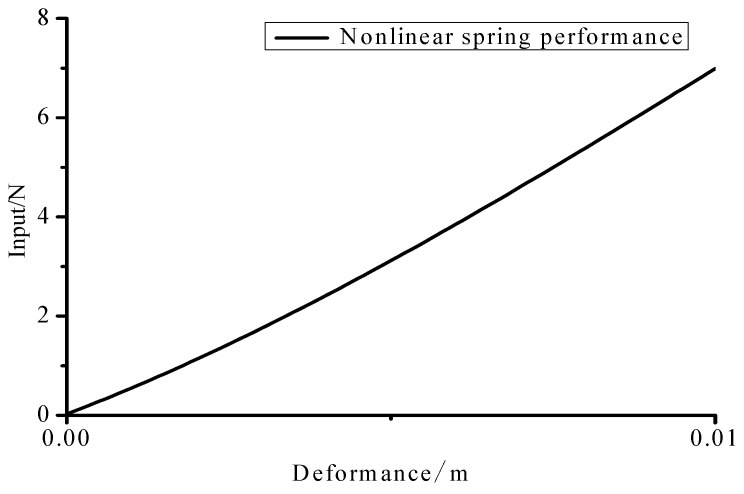
Nonlinear spring performance.

Considering a cantilever with a nonlinear spring stalled at the end node, the finite element model and the FBG (Fiber Bragg Grating) sensing network are shown in Figure 12. In the experiment, the strain values of FBG sensor network were used as observed values. The parameters of the beam are: Elastic modulus $E=6.89\times 10^{10}\;\left(N/m^{2}\right);$ Density $\rho =2.69\times 10^{3}\;\left(\mathrm{kg}/m^{3}\right)$; Beam length l=0.48 m; Cross section S=0.03 m×0.003 m. The performance of the nonlinear spring is plotted in Figure 13. There were six measuring points of FBG on the beam along its center line which were employed to record the beam’s surface strain simultaneously. The distance between two consecutive sensors was about 9.15 cm.

The relationship between wavelength shift of FBG sensors and strain values is showed in Equation (33). Supposing with no temperature change, the strain value can be computed by measuring the wavelength change according to Equation (34):

(33) $$\Delta \lambda =\lambda [(1-P_{e})\varepsilon +(a+\xi )\Delta T]$$

(34) $$\varepsilon =\frac{1}{P_{e}}\frac{\Delta \lambda }{\lambda }$$

where Δλ is the wavelength shift, ε is the axial strain, α is the thermal expansion coefficient, ξ is the thermo-optical coefficient, ΔT is the temperature change, and $P_{e}$ is the effective photo-elastic coefficient [19,20,21].

For a Euler-Bernoulli beam with n-element, the relationship between strain values and nodal degrees of freedom can be described as follows:

(35) $$\left[\begin{array}{l} \varepsilon_{1}\\ \varepsilon_{2}\\ \vdots \\ \vdots \\ \varepsilon_{2n-1}\\ \varepsilon_{2n} \end{array}\right]=\left[\begin{array}{cccccccc} B_{1} & & & & & & & \\ B_{2} & & & & & & & \\ & & B_{3} & & & & & \\ & & B_{4} & & & & & \\ & & & & \ddots & & & \\ & & & & & \ddots & & \\ & & & & & & & B_{2n-1}\\ & & & & & & & B_{2n} \end{array}\right]\left[\begin{array}{l} w_{1}\\ \theta_{1}\\ w_{2}\\ \theta_{2}\\ \vdots \\ w_{n-1}\\ \theta_{n-1}\\ w_{n}\\ \theta_{n} \end{array}\right]$$

where $\delta ={\left[w_{i},\theta_{i,}w_{j},\theta_{j}\right]}^{T}$, $B(\xi )=\frac{1}{l^{2}}[-6+12\xi ,l(-4+6\xi ),6-12\xi ,l(-2+6\xi )]\times \frac{h}{2}$. B(ξ) is the shape function; l is the length of the beam element; ξ=x/l, *x* is the location of the FBG in element; *h* is the thickness of the beam; w is nodal displacement; θ is nodal rotation. ${\left[\varepsilon_{1},\varepsilon_{2},\cdots \varepsilon_{2n}\right]}^{T}$ is strain values vector. For the experiment with six FBG sensors pasted on beam, observation matrix can be describes as follow:

(36) $$H=\left[\begin{array}{cccccc} B_{1} & & & & & \\ B_{2} & & & & & \\ & & B_{3} & & & \\ & & B_{4} & & & \\ & & & & & B_{5}\\ & & & & & B_{6} \end{array}\right]$$

### 4.2. Experimental Procedure and Results

**Figure 14:**
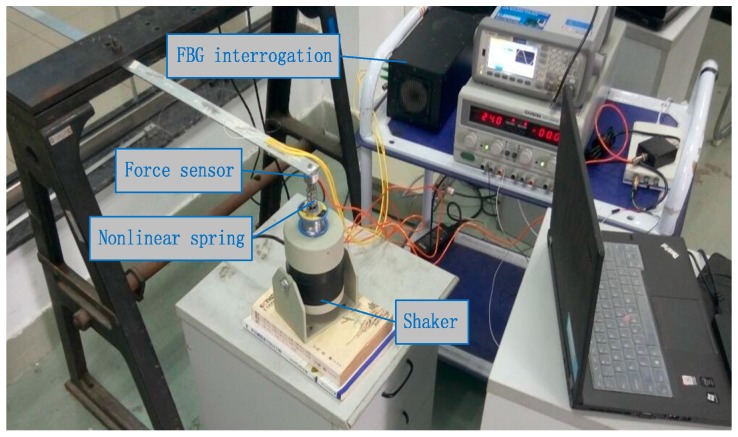
Layout of experiment.

**Figure 15:**
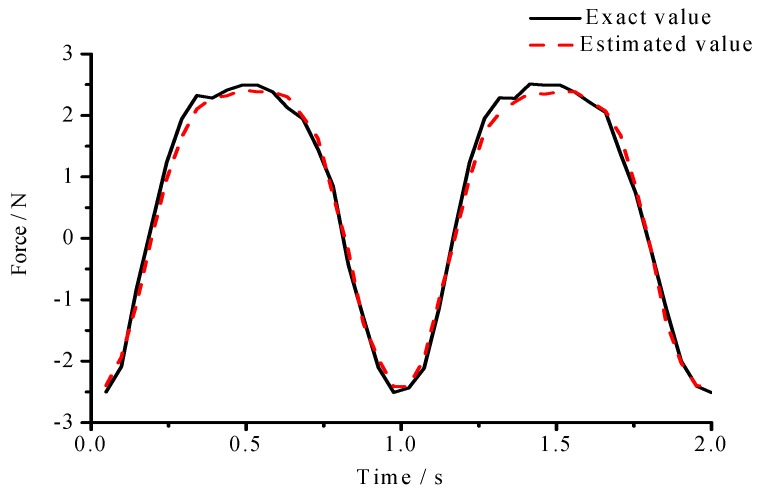
Experiment results of input estimation.

The layout of experiment is plotted in Figure 14. FBG (Fiber Bragg Grating) interrogation system (SM130 (SonMicro Elektronik, Mersin, Turkey)) is used for measuring the dynamic strains, and an electrodynamics shaker is employed for the excitation. Input force is applied at the beam end, and the magnitude and location of input force are estimated by FBG sensor network. In the experiment, a force sensor is stalled between the exciter and beam end, and the measured force is used as an exact value to verify the practicability of the proposed method. In the process, a NI cDAQ-9174 module (National Instruments Corporation, Austin, TX, USA) and LABVIEW software (National Instruments Corporation, Austin, TX, USA) were used to acquire the signal. The sampling frequency was set as 100 Hz, and the experimental time is was two seconds. The results of input force estimation were plotted in Figure 15.

### 4.3. Discussions

(1) Experimental results showed that the estimated input forces had a little amplitude error. The amplitude error was mainly produced by insufficient number of sensors. In engineering applications, distributed optical fiber sensors network can solve the deficiency of sensors installation, where the distance between two consecutive sensors can be less than 1 cm.

(2) The proposed estimation method required the statistical characteristics of noise to be known, as well as an accurate system model. In practice, sometimes the statistical characteristics of noise are unknown, and the system model is inaccurate. In addition, the nonlinear system is easily affected by the model uncertainties in the actual operating environment. For these deficiencies, we employed an adaptive algorithm to estimate the time-varying noise statistics and model uncertainties.

## 5. Conclusions

A real-time nonlinear method for estimating input forces is presented in this work. The method used SRCKF to suppress noise and a nonlinear estimator to estimate input forces. Simulations of the large deflection beam system and experiment of a linear beam constrained by a nonlinear spring were applied. Simulation results showed that the mean errors and RMSE values of three types of input forces subjected to the above noise were satisfied. Experimental results showed that the estimated input forces had a little amplitude error, and the estimation method had good stability.

## Figures and Tables

**Table 1:** Estimation performance of three types forces with varying *Q_w_* (*σ* = 1 × 10^−8^).

Force Type	Sinusoidal		Rectangular		Triangular	
$Q_{w}$	1 × $10^{-4}$	1 × $10^{-6}$	1 × $10^{-4}$	1 × $10^{-6}$	1 × $10^{-4}$	1 × $10^{-6}$
Mean ($10^{-4}$)	9.61	1.72	60.1	50.1	25.5	16.4
RMSE (%)	0.46	0.43	6.61	5.41	5.27	3.62

## Appendix A

Following the nonlinear discrete system as given in:

(A1) $$X_{k}=f(X_{k-1},F_{k-1})+w_{k}$$

(A2) $$Z_{k}=h(X_{k})+v_{k}$$

where *w*(*k*) and *v*(*k*) are white noise sequences, $E[w_{k}w_{j}]=Q_{k}\delta_{k,j},E[v_{k}v_{j}]=\sigma_{k}{}^{2}\delta_{k,j}$.

The posteriori state estimate without the exciting force:

(A3) $$\begin{array}{ll} {\bar{X}}_{k} & =f({\bar{X}}_{k-1})+K_{k}[Z_{k}-h(f({\bar{X}}_{k-1}))]\\ & =f({\bar{X}}_{k-1})-K_{k}h(f({\bar{X}}_{k-1}))+K_{k}Z_{k} \end{array}$$

The posteriori state estimate with the exciting force:

(A4) $$\begin{array}{ll} {\hat{X}}_{k} & =f({\hat{X}}_{k-1},F_{k-1})+K_{k}[Z_{k}-h(f({\hat{X}}_{k-1},F_{k-1}))]\\ & =f({\hat{X}}_{k-1},F_{k-1})-K_{k}h(f({\hat{X}}_{k-1},F_{k-1}))+K_{k}Z_{k}\\ & =f({\hat{X}}_{k-1},0)+\Gamma_{k}F_{k-1}+K_{k}Z_{k}-K_{k}h(f({\hat{X}}_{k-1},0)+\Gamma_{k}F_{k-1})\\ & =f({\hat{X}}_{k-1},0)+\Gamma_{k}F_{k-1}+K_{k}Z_{k}-K_{k}(h(f({\hat{X}}_{k-1},0))+\Phi_{k}\Gamma_{k}F_{k-1}) \end{array}$$

where $K_{k}$ is got from SRCKF, and:

$$\Phi_{k}=\partial f(\hat{X}(k|k-1))/\partial X\;\;\Gamma_{k}=\partial f(\hat{X}(k|k-1))/\partial F\;\;H_{k}=\partial h(\hat{X}(k|k-1))/\partial X$$

Define the difference of the two posteriori state estimate as follows:

(A5) $$\begin{array}{l} \Delta X_{k}={\hat{X}}_{k}-{\bar{X}}_{k}\\ \;\;=(I-K_{k}H_{k})\Phi_{k}({\hat{X}}_{k-1}-{\bar{X}}_{k-1})+(I-K_{k}H_{k})\Gamma_{k}F_{k-1} \end{array}$$

Assume the exciting force begin with the time $t_{n}$, then:

$$1\;k\;<\;n.\;{\hat{X}}_{k-1}-{\bar{X}}_{k-1}\;=\;0.\;F_{k-1}\;=\;0,\;\mathrm{so}\;\Delta X_{k}\;=\;0$$

$$2\;k\;=\;n.\;{\hat{X}}_{k-1}-{\bar{X}}_{k-1}\;=\;0.F_{k-1}\;=\;0,\;\mathrm{so}\;\Delta X_{k}\;=\;0$$

$$3\;k\;>\;n.\;{\hat{X}}_{k-1}-{\bar{X}}_{k-1}=\Delta X_{k-1}$$

so

(A6) $$\begin{array}{ll} \Delta X_{k} & =(I-K_{k}H_{k})\Phi_{k}({\hat{X}}_{k-1}-{\bar{X}}_{k-1})+(I-K_{k}H_{k})\Gamma_{k}F_{k-1}\\ & =(I-K_{k}H_{k})(\Phi_{k}\Delta X_{k-1}+\Gamma_{k}F_{k-1}) \end{array}$$

In summary, we get:

(A7) $$\Delta X_{k}=\left\{ \begin{array}{ll} 0 & k\leq n\\ \left(I-K_{k}H_{k})(\Phi_{k}\Delta X_{k-1}+\Gamma_{k}F_{k-1}\right) & k>n \end{array}\right.$$

At the time $t_{n+1}$, Equation (A7) becomes:

(A8) $$\Delta X_{n+1}=(I-K_{n+1}H_{n+1}){\left(\Phi_{n+1}\Delta X_{n}+\Gamma_{n+1}F_{n}\right)}_{}$$

From (A7) we know that $\Delta X_{n}$ = 0, so Equation (A8) becomes:

(A9) $$\Delta X_{n+1}=(I-K_{n+1}H_{n+1})\Gamma_{n+1}F_{n}$$

Define:

$$M_{n+1}=I-K_{n+1}H_{n+1}$$

Then Equation (A9) becomes:

(A10) $$\Delta X_{n+1}=M_{n+1}\Gamma_{n+1}F_{n}$$

From Equations (A7) and (A10), for *k* > *n*, we have:

(A11) $$\Delta X_{k}=(I-K_{k}H_{k})(\Phi_{k}\Delta X_{k-1}+\Gamma_{k}F_{k-1})$$

Ignoring $\Phi_{k}\Delta X_{k-1}$, and Equation (A11) becomes:

(A12) $$\Delta X_{k}=M_{k}\Gamma_{k}F_{k-1}$$

From Equations (A12) and (A11), we have:

$$\begin{array}{cc} M_{k}\Gamma_{k}F_{k-1} & =(I-K_{k}H_{k})(\Phi_{k}\Delta X_{k-1}+\Gamma_{k}F_{k-1})\\ & =(I-K_{k}H_{k})\Phi_{k}M_{k-1}\Gamma_{k-1}F_{k-2}+(I-K_{k}H_{k})\Gamma_{k}F_{k-1} \end{array}$$

Assume $F_{k-1}$ = $F_{k-2}$, then:

(A13) $$M_{k}=(I-K_{k}H_{k})(\Phi_{k}M_{k-1}+I)$$

From Equations (A12) and (A13), we have:

(A14) $${\hat{X}}_{k}={\bar{X}}_{k}+M_{k}\Gamma_{k}F_{k-1}$$

where:

$$M_{k}=\left\{ \begin{array}{ll} 0 & k\leq n\\ \left(I-K_{k}H_{k})(\Phi_{k}M_{k-1}+I\right) & k>n \end{array}\right.$$

The observed value of the residual sequence with exciting force can be described as:

(A15) $${\hat{Z}}_{k}=Z_{k}-h(f({\hat{X}}_{k-1},F_{k-1}))$$

The observed value of the residual sequence without exciting force can be described as:

(A16) $${\bar{Z}}_{k}=Z_{k}-h(f({\bar{X}}_{k-1}))$$

For different values of *k*, we have:

$$1\;k\;<\;n.\;F_{k-1}\;=\;0,{\bar{Z}}_{k}={\hat{Z}}_{k}$$

$$2\;k\;=\;n.\;F_{k-1}\;=\;0,{\bar{Z}}_{k}={\hat{Z}}_{k}$$

$$3\;k\;>\;n.\;F_{k-1}\neq 0,\;{\bar{Z}}_{k}\neq {\hat{Z}}_{k}$$

$$\begin{array}{ll} {\bar{Z}}_{k}-{\hat{Z}}_{k} & =H_{k}\Phi_{k}({\hat{X}}_{k-1}-{\bar{X}}_{k-1})+H_{k}\Gamma_{k}F\\ & =H_{k}(\Phi_{k}M_{k-1}+I)\Gamma_{k}F \end{array}$$

In summary, we get:

(A17) $${\bar{Z}}_{k}=\left\{ \begin{array}{ll} {\hat{Z}}_{k} & k\leq n\\ {\hat{Z}}_{k}+B_{k}F & k>n \end{array}\right.$$

where:

$$B_{k}=H_{k}(\Phi_{k}M_{k-1}+I)\Gamma_{k}$$

For *k* = *n* + 1, *n* + 2, …, *n* + *l*. we have:

(A18) Y=ψF+ε

where:

$$Y(N)=\left[\begin{array}{l} {\bar{Z}}_{n+1}\\ {\bar{Z}}_{n+2}\\ \vdots \\ {\bar{Z}}_{n+l} \end{array}\right]\;\;\varepsilon (N)=\left[\begin{array}{l} {\hat{Z}}_{n+1}\\ {\hat{Z}}_{n+2}\\ \vdots \\ {\hat{Z}}_{n+l} \end{array}\right]\;\;\psi (N)=\left[\begin{array}{l} B(n+1)\\ B(n+2)\\ \vdots \\ B(n+l) \end{array}\right]=\left[\begin{array}{l} H_{n+1}\Gamma_{n+1}\\ H_{n+2}(\Phi_{n+2}M_{n+1}+I)\Gamma_{n+2}\\ \vdots \\ H_{n+l}(\Phi_{n+l}M_{n+l-1}+I)\Gamma_{n+l} \end{array}\right]$$

$$M_{n+l}=\left\{ \begin{array}{ll} 0 & l=0\\ \left(I-K_{n+l}H_{n+l})[\Phi_{n+l}M_{n+l-1}+I\right] & l>0 \end{array}\right.$$

Assume $E[\hat{z}(k){\hat{z}}^{T}(k)]=s(k)$, s(k) is got from UKF. ε(N) is a disturbance vector, and its variance is given by:

(A19) $$\Sigma (N)=\left[\begin{array}{cccc} s(n+1) & 0 & \ldots & 0\\ 0 & s(n+2) & \ldots & 0\\ & & \ddots & \\ 0 & 0 & \cdots & s(n+l) \end{array}\right]$$

From Equation (A18), we can get:

(A20) $$\hat{F}(N)={\left[\psi^{T}(N)\Sigma^{-1}(N)\psi (N)\right]}^{-1}\psi^{T}(N)\Sigma^{-1}(N)Y(N)$$

And error covariance matrix is:

(A21) $$\begin{array}{ll} P_{b}(N) & =E\left\{\left[F-\hat{F}(N)\right]{\left[F-\hat{F}(N)\right]}^{T}\right\}\\ & ={\left[\psi^{T}(N)\Sigma^{-1}(N)\psi (N)\right]}^{-1} \end{array}$$

Including forgetting factor *γ*, from (A19), we get:

(A22) $$\Sigma^{-1}(N)=\left[\begin{array}{cccc} s^{-1}(n+1)\gamma^{l-1} & 0 & \ldots & 0\\ 0 & s^{-1}(n+2)\gamma^{l-2} & \ldots & 0\\ & & \ddots & \\ 0 & 0 & \cdots & s^{-1}(n+l) \end{array}\right]$$

For *k* = *n* + 1, from Equations (A17), (A18) and (A22), we have:

(A23) $$\bar{Z}(N+1)=B(N+1)F+\hat{Z}(N+1)$$

(A24) Y(N+1)=ψ(N+1)F(N+1)+ε(N+1)

(A25) $$\Sigma^{-1}(N+1)=\left[\begin{array}{cc} \gamma \Sigma^{-1}(N) & 0\\ 0 & s^{-1}(N+1) \end{array}\right]$$

where:

$$Y(N+1)=\left[\begin{array}{l} Y(N)\\ \bar{Z}(N+1) \end{array}\right]\;\;\psi (N+1)=\left[\begin{array}{l} \psi (N)\\ B(N+1) \end{array}\right]\;\;\varepsilon (N+1)=\left[\begin{array}{l} \varepsilon (N)\\ \hat{Z}(N+1) \end{array}\right]$$

From Equations (A20) and (A21), we have:

(A26) $$\begin{array}{ll} \hat{F}(N+1) & ={\left[\psi^{T}(N+1)\Sigma^{-1}(N+1)\psi (N+1)\right]}^{-1}*\psi^{T}(N+1)\Sigma^{-1}(N+1)Y(N+1)\\ & ={\left[\gamma \psi^{T}(N)\Sigma^{-1}(N)\psi (N)+B^{T}(N+1)s^{-1}(N+1)B(N+1)\right]}^{-1}*\left[\gamma \psi^{T}(N)\Sigma^{-1}(N)Y(N)+B^{T}(N+1)s^{-1}(N+1)\bar{Z}(N+1)\right] \end{array}$$

(A27) $$\begin{array}{ll} P_{b}(N+1) & ={\left[\psi^{T}(N+1)\Sigma^{-1}(N+1)\psi (N+1)\right]}^{-1}\\ & ={\left[\gamma P_{b}{}^{-1}(N)+B^{T}(N+1)s^{-1}(N+1)B(N+1)\right]}^{-1} \end{array}$$

Substituting Equation (A21) into Equation (A27), we have:

(A28) $$P_{b}(N+1)=\gamma^{-1}P_{b}(N)-\gamma^{-1}P_{b}(N)B^{T}(N+1)*{\left[B(N+1)\gamma^{-1}P_{b}(N)B^{T}(N+1)+s(N+1)\right]}^{-1}\begin{array}{c} {}*B(N+1)\gamma^{-1}P_{b}(N) \end{array}$$

Substituting Equation (A20) into Equation (A26), we have:

(A29) $$\begin{array}{ll} \hat{F}(N+1)= & \hat{F}(N)+\gamma^{-1}P_{b}(N)B^{T}(N+1)s^{-1}(N+1)\bar{Z}(N+1)-\gamma^{-1}P_{b}(N)B^{T}(N+1)*\\ & \left[{\left[B(N+1)\gamma^{-1}P_{b}(N)B^{T}(N+1)+s(N+1)\right]}^{-1}B(N+1)\right]\left[\hat{F}(N)+\gamma^{-1}P_{b}(N)B^{T}(N+1)s^{-1}(N+1)\bar{Z}(N+1)\right] \end{array}$$

We insert the following term between $B^{T}(N+1)$ and $s^{-1}(N+1)$, and we will get the outcomes:

$${\left[B(N+1)\gamma^{-1}P_{b}(N)B^{T}(N+1)+s(N+1)\right]}^{-1}\begin{array}{cc} {}* & \left[B(N+1)\gamma^{-1}P_{b}(N)B^{T}(N+1)+s(N+1)\right] \end{array}$$
